# A duplex nested RT-PCR method for monitoring porcine epidemic diarrhea virus and porcine delta-coronavirus

**DOI:** 10.1186/s12917-023-03708-y

**Published:** 2023-09-08

**Authors:** Chun Qi Li, Li Qun Hu, Guo Ping Liu, Yan Wang, Tong Li, Shao Xian Chen, Xiao Lin Yang, Li Xin Ma, Jian Guo Zeng

**Affiliations:** 1 College of Animal Science, Yangtze University, Jingzhou, China; 2 State Key Laboratory of Biocatalysis and Enzyme Engineering, Hubei Key Laboratory of Industrial Biotechnology, School of Life Sciences, Hubei University, Wuhan, China; 3 Center for Disease Control and Prevention of Xinzhou Distract, Wuhan, China; 4 Hunan Key Laboratory of Traditional Chinese Veterinary Medicine, Hunan Agricultural University, Changsha, Hunan China

**Keywords:** Porcine epidemic diarrhea virus, Porcine delta-coronavirus, Duplex nested RT-PCR, Clinical monitoring

## Abstract

**Background:**

Porcine epidemic diarrhea virus (PEDV) and porcine delta-coronavirus (PDCoV) are economically important pathogens that cause diarrhea in sows and acute death of newborn piglets. Moreover, the emerging PDCoV was reported to infect children. The current situation is that vaccine prevention has not met expectations, and emergency containment strategies following outbreaks cannot prevent the damages and losses already incurred. Therefore, a more sensitive detection method, that is both convenient and enables accurate and effective sequencing, that will provide early warning of PEDV and PDCoV is necessary. This will enable active, effective, and comprehensive prevention and control, which will possibly reduce disease occurrences.

**Results:**

Duplex nested RT-PCR (dnRT-PCR) is an ideal method to achieve early warning and monitoring of PEDV and PDCoV diseases, and to additionally investigate any molecular epidemiological characteristics. In this study, two pairs of primers were designed for each virus based upon the highly conserved N protein sequences of both PEDV and PDCoV strains retrieved from the NCBI Genbank. After optimization of the reaction conditions, the dnRT-PCR assay amplified a 749-bp fragment specific to PEDV and a 344-bp fragment specific to PDCoV. Meanwhile, the specificity and sensitivity of the primers and clinical samples were tested to verify and establish this dnRT-PCR method. The limit of detection (LoD)for both PEDV and PDCoV was 10 copies/µL. The results showed that among 251 samples, 1 sample contained PEDV infection, 19 samples contained a PDCoV infection, and 8 samples were infected with both viruses, following the use of dnRT-PCR. Subsequently, the positive samples were sent for sequencing, and the sequencing results confirmed that they were all positive for the viruses detected using dnRT-PCR, and conventional RT-PCR detection was conducted again after the onset of disease. As these results were consistent with previous results, a detection method for PEDV and PDCoV using dnRT-PCR was successfully established. In conclusion, the dnRT-PCR method established in this study was able to detect both PEDV and PDCoV, concomitantly.

**Conclusions:**

The duplex nested RT-PCR method represents a convenient, reliable, specific, sensitive and anti-interference technique for detecting PEDV and PDCoV, and can additionally be used to simultaneously determine the molecular epidemiological background.

**Supplementary Information:**

The online version contains supplementary material available at 10.1186/s12917-023-03708-y.

## Background

Swine acute diarrhea syndrome(SADS)is one of the most serious diseases affecting the swine industry in China and the rest of the world in recent years [1–3]. Some prevention and control measures have been undertaken to combat this disease [4, 5]. Although these interventions have achieved some successful outcomes, they are far from being effective or even efficient regarding prevention and control. Comprehensive measures are usually taken after an outbreak of SADS, which has caused significant economic losses [6–8]. Porcine epidemic diarrhea virus (PEDV) and porcine deltacoronavirus (PDCoV) are the main causative agents of PDS, and limited approaches have been developed to prevent and control these two virues. The main clinical symptoms of SADS include are acute diarrhea in sows and fattening pigs, severe vomiting, diarrhea and dehydration in newborn piglets with high death rate [9–11]. In addition to diarrhea, SADs also increased the abortion and premature delivery rates in some sows.

Porcine epidemic diarrhea (PED) is an acute intestinal infectious disease in pigs characterized by watery diarrhea, vomiting, and dehydration [8, 12], commonly known as winter diarrhea disease. PEDV belongs to the family Coronaviridae, genus Alphacoronavirus, and contains an infectious single-stranded, positive-sense RNA genome (nearly 28 kb) [13, 14]. The PEDV genome contains six open reading frames (ORFs) and encodes the replicase polymeric protein 1ab (pp1ab), spike (S), envelope (E), membrane (M) and nucleocapsid (N) from 5’-3’ [15].

PDCoV also belongs to the member of the Coronaviridae family, genus deltacoronavirus [16]. Diarrhea outbreaks in newborn piglets have occurred frequently in pig farms across China since early winter 2010. Previously, PEDV was generally considered to be the main pathogenic pathogen in these cases. Subsequently, another novel coronavirus, PDCoV, was found to cause diarrhea in piglets and has become widespread in pig farms in China. As an emerging porcine intestinal coronavirus, PDCoV was first reported in Hong Kong, China in 2012 [17], and later in the USA [18], China Mainland [19], South Korea [20], and Thailand [21]. The novel porcine enteric coronavirus disease can cause diarrhea and vomiting in suckling pigs [22], with morbidity and mortality rates as high as 50-100%, and low mortality post-infection in growing and adult pigs. In addition, from May 2014 to December 2015, Lednicky et al. collected a total of 369 samples from children presenting to the school clinic with acute undifferentiated febrile illness in Haiti and found three of them were infected with coronavirus strains, which were further identified as PDCoV [23]. Therefore, it means that PDCoV also poses a potential threat to human health.

PDCoV is an enveloped virus that has a positive-sense single-stranded RNA (+ ssRNA) genome measuring 25.4 kb in length [24, 25]. The PDCoV genome has seven major open reading frames (ORFs). Two overlapping ORFsc(ORF1a and ORF1b) which encode two replication-associated proteins, both are autoproteolytically cleaved into 15 nonstructural proteins (Nsp2 to Nsp16) [26, 27]. The remaining ORFs encodes the S, E, M and N. Additionally, three accessory proteins have been identified: nonstructural protein 6 (NS6), NS7, and NS7a [28–30].

Genetically, the N genes are highly conserved compared with the E, S, and M genes of PEDV and PDCoV [24], therefore the N gene is a suitable target to develope molecular diagnostic approach, such as RT-PCR and fluorescence quantitative RT-PCR [31, 32]. Moreover, the co-infection of PEDV and PDCoV is common now, so it is necessary to establish duplex RT-PCR.

In view of the urgent situation in the swine industry, a method needs to be established with three main attributes, all of which have been addressed in the present research. First of all, sensitivity of the method must be particularly high. This would enable monitoring, analysis and prediction of the disease prior to outbreak, so as to achieve the role of an early warning system. Secondly, using the two pathogens detailed in this study, both could be monitored and used as an early warning system or for diagnosis simultaneously. Finally, positive samples could ben be sequenced, and this information used immediately to clarify the genotype and molecular evolution characteristics of the infection or outbreak. Although the positive products can be sent for sequencing following the presently used RT-PCR monitoring methods, sensitivity is relatively low. In addition, although the sensitivity of fluorescence quantitative monitoring is relatively good, the process of testing and sequencing is relatively cumbersome. Therefore, in this study, on the basis of conventional RT-PCR, nuclear protein N was used as the diagnostic target gene to establish a dnRT-PCR method that could detect both PEDV and PDCoV simultaneously, so as to improve the sensitivity and specificity of detection. Therefore, the method described in this study has the three basic attributes mentioned above, and additionally satisfies the industry goal of moving from post-onset diagnosis through to pre-onset warning.

As mentioned above, newborn piglets aged 3-5 days have a high incidence of vomiting and diarrhea clinically. It is possible that toxic sow milk leads to cumulative infection of newborn piglets, and if sows have post-mating estrus or an abortion, it may also be related to PEDV and PDCoV. In this study, based on pathogen detection in sow vulva swabs and colostrum via dnRT-PCR, correlations were determined. Therefore, it is possible to use the method outlined in this study to monitor colostrum and vulva swab samples, so as to achieve early warning, and to synchronously identify the etiology of the two pathogens in advance of serious infection and outbreak.

## Results

### Specificity of the dnRT-PCR

**Fig. 1:**
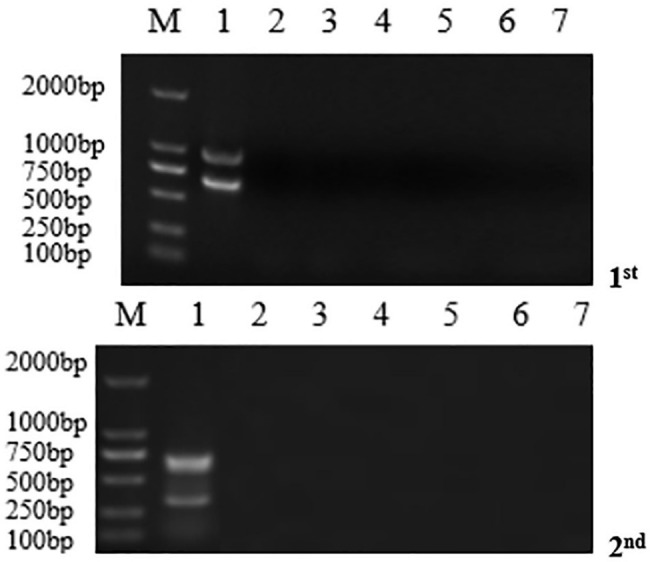
Gel electrophoresis of the duplex nested RT-PCR for outer and internal primer specificity detection. M, DL2000 DNA Marker; lane 1, recombinant positive plasmid; lane 2, PRRSV; lane 3, TGEV; lane 4, PoRV; lane 5, PRV; lane 6, Escherichia coli (E-coli) and Staphylococcus aureus (S. aureus); lane 7, negative control (RNase-free water)

The specificity of the dnRT-PCR was evaluated for target organism using nested primers. First, the specificity was tested using a recombinant positive plasmid. Second, other pathogens associated with diarrheal disease of pigs, including PRRSV, TGEV, PoRV, PRV, Escherichia coli and Staphylococcus aureus, were tested using the dnRT-PCR assay to evaluate its analytical specificity. The detection results showed that there was no amplification product for the negative control or the pathogens in the first reaction, and there was also no band for the second reaction, which proved that the four pairs of primers had good specificity and exhibited high amplification efficiency (Fig. 1).

### Sensitivity of dnRT-PCR

**Fig. 2:**
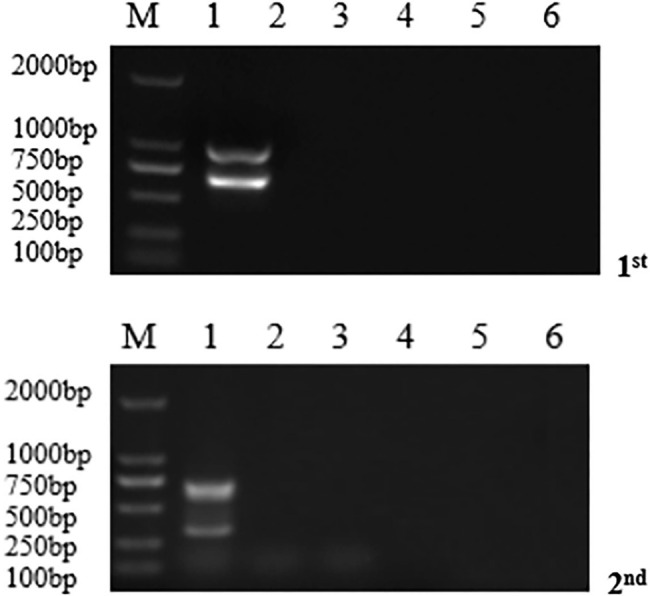
Comparison of sensitivity among the newly developed duplex nested RT-PCR and duplex RT-RPA as described in previously published papers for PEDV, PDCoV, and a mixture of the two viruses. The numbers at the top indicate the orders of the gene copy number of virus. The dividing line divides the figures from the different gels

The sensitivity results from the dnRT-PCR and duplex RT-PRA were shown in Fig. 2. The electrophoresis results from the first PCR amplification showed that the copy number of the gene detected by the outer primer was 10^2^ copies/uL, and the amplification specificity was good. The minimum gene copy number of 10^1^ orders of magnitude was detected via double nested RT-PCR amplification. The sensitivity of this method was 1 order of magnitude higher than that of duplex RT-RPA.

### Interference test

**Fig. 3:**
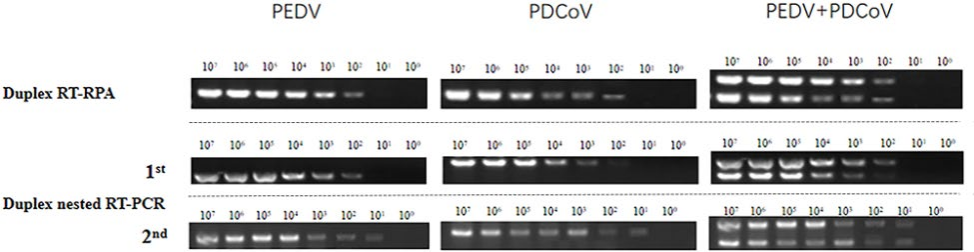
Gel electrophoresis of interference detection by the duplex nested RT-PCR primers, M, DL2000 DNA Marker; lane 1, the mixed genomes were amplified by outer and internal primers respectively; lane 2–6, negative control (RNase-free water)

The detection results showed that all four pairs of primers were able to amplify specific bands from the mixed template, which showed that the primers exhibited good anti-interference performance (Fig. 3).

### Evaluation of dnRT-PCR on clinical samples

**Fig. 4:**
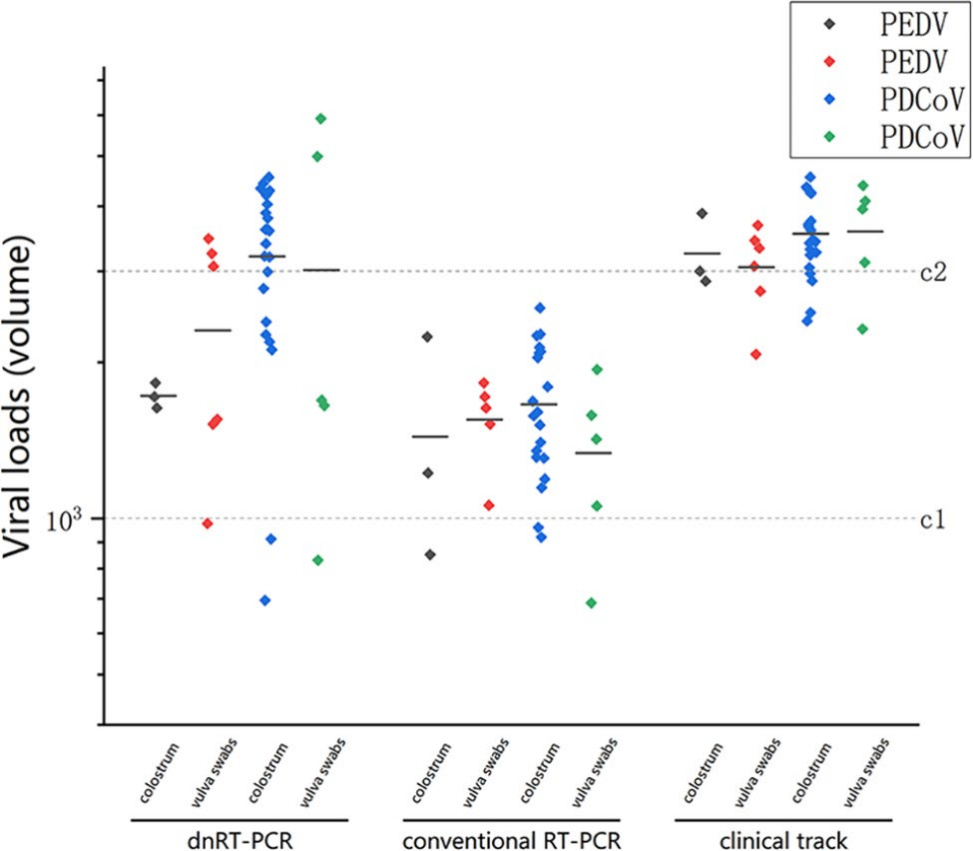
Two methods were used to detect the viruses and the coincidence rates of the results were compared the clinical track results recorded at a later time point. The lower broken line indicates the cutoff value (c1 = 1000) and the upper line depicts the twofold cutoff value (c2 = 4000). Based on the volume, the viral loads were classified into three levels: low viral load (volume ≤ c1), medium viral load (c1 < volume ≤ c2) and high viral load (volume > c2)

As shown in Fig. 4, the detection results using the dnRT-PCR method were consistent with those observed in the sequencing and clinical track. Among these samples, one sample was identified as containing a pure infection of PEDV, nineteen samples had only PDCoV present, and eight samples (three colostrum and five vulva) had both PEDV and PDCoV identified using the dnRT-PCR (Table 1). In contrast, using conventional RT-PCR one positive sample was identified as a PEDV only infection, but only eighteen had PDCoV identified and only seven samples contained both PEDV and PDCoV, the latter of which included three colostrum samples and just 4 vulva samples (Table 1). The above results indicated that one positive sample of PEDV and two positive samples of PDCoV were not detected using conventional RT-PCR, which may be attributed to the low viral load within those particular samples, indicating an infection of early stage, which is difficult to detect by conventional RT-PCR.

**Table 1:** Concordance rate comparisons of the dnRT-PCR with the duplex RT-RPA for the detection of clinical specimens

Detection method	Sample type	No. of Samples	No. of positive samples (positive %)		
			PEDV	PDCoV	Coinfection
dnRT-PCR	Colostrum	177	3 (1.2)	22 (8. 8)	3 (1. 7)
	Vulva swab	74	6 (2. 4)	5 (2. 0)	5 (2. 0)
	Total	251	9 (3. 6)	27 (10. 8)	8(3. 2)
conventional RT-PCR	Colostrum	177	3 (1. 2)	20 (8. 0)	3 (1. 7)
	Vulva swab	74	5 (2. 0)	5 (2. 0)	4(1. 6)
	Total	251	8 (3. 2)	25 (10. 0)	7(2. 8)

Note: Positive rate = number of positive samples/total number of tested samples

### Nucleotide homology analysis of the N gene sequence in PEDV and PDCoV

**Fig. 5:**
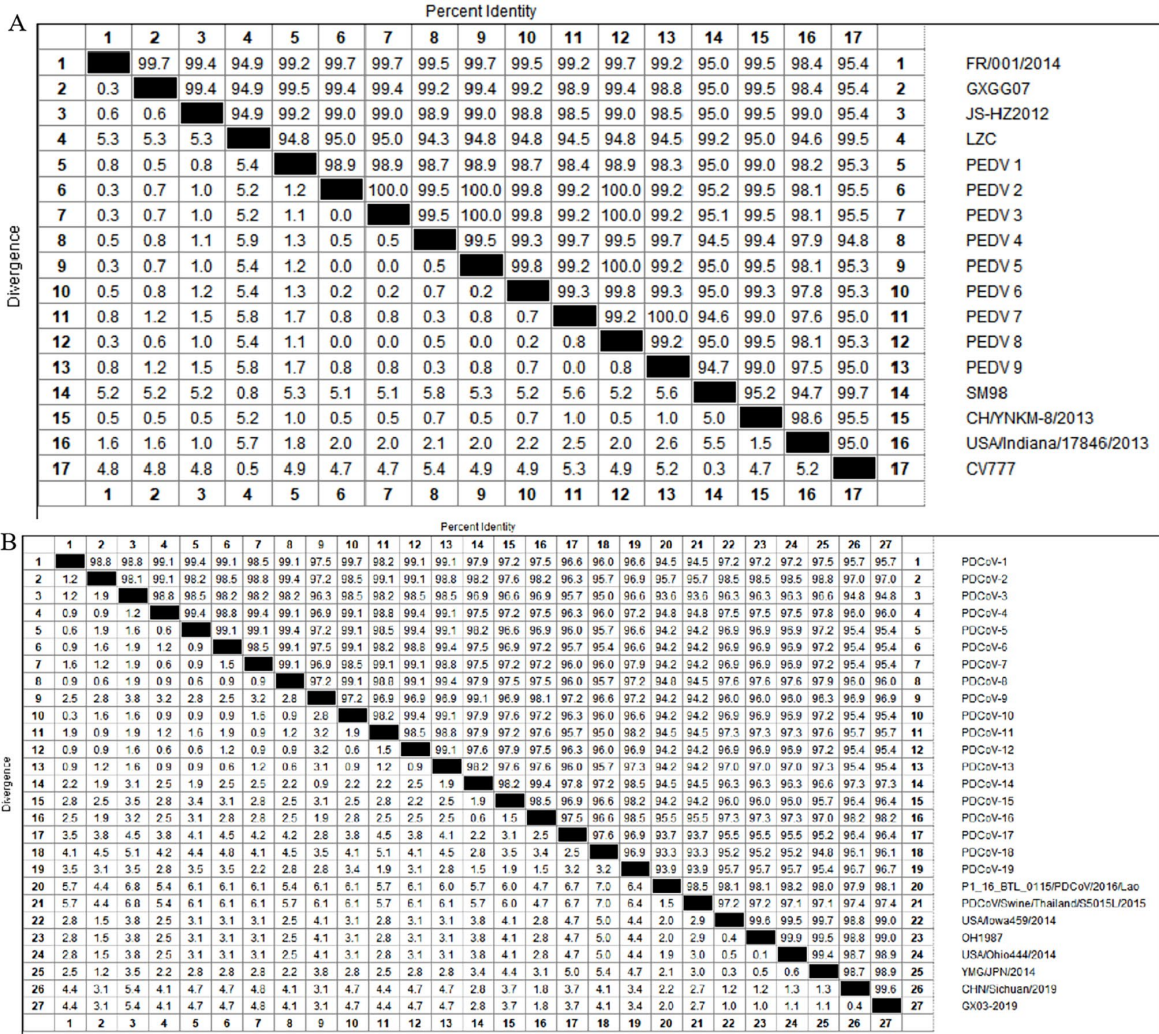
A Comparison of nucleotide homology of the N gene in the 9 sequencing PEDV strains from this paper and 8 PEDV reference strains. B Comparison of nucleotide homology of the N gene using 19 sequencing PDCoV strains from this paper and 8 PDCoV reference strains

MegAlign software(MegAlign Pro | Sequence Alignment Software | DNASTAR) was used to compare the nucleotide homology of N gene sequences in 9 sequenced PEDV strains with locus 216–956 and 8 PEDV reference strains, and the N gene sequence of 19 sequencing PDCoV representative strains with locus 440–784 and 8 PDCoV reference strains, respectively (Fig. 5). The results showed that the PEDV strains showed high homologies, ranging from 94.3 to 100%, and the homology of the PDCoV strains also exhibited high homologies, ranging from 93.3 to 99.9%. Therefore, the accuracy of the dnRT-PCR method was further confirmed by the homology analysis undertaken against the sequencing strains in this study.

### Genetic evolution tree analysis of N gene sequence in PEDV and PDCoV

**Fig. 6:**
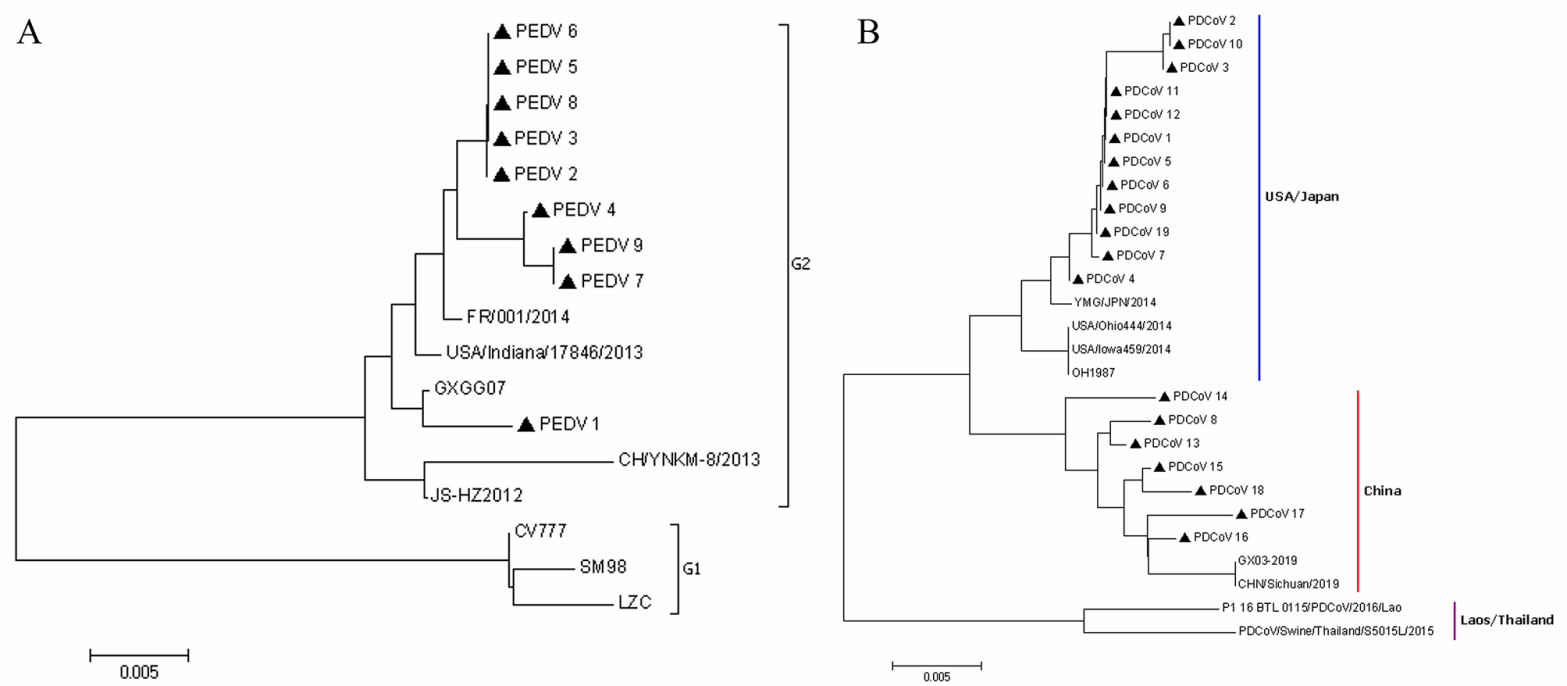
A Genetic evolution tree analysis of N gene sequence of PEDV strains. ▲represents the strains obtained in this study. B Genetic evolution tree analysis of N gene sequence of PDCoV strains. ▲represents the strains obtained in this study

MEGA 7.0 software was used to draw the Genetic evolution tree of N gene of 9 PEDV strains in this study and 8 PEDV representative strains at home and abroad (Fig. 6A). The 17 PEDV sequences mainly formed three evolutionary branches, G1 and G2. The G1 branch was mainly composed of CV777, SM98 and LZC strains, while the G2 branch was composed of FR/001/2014, USA/Indiana/17,846/2013, GXGG07, JS-HZ2012 and CH/YNKM-8/2013 representative strains. The strains in this study belongs to the G2 branch and are close to the genetic evolution of the FR/001/2014 and GXGG7 strains.

MEGA 7.0 software was also used to draw the Genetic evolution tree of N gene of 19 PDCoV representative strains in this study and 8 PDCoV representative strains at home and abroad (Fig. 6B). PDCoV was classified into three major lineages, USA/Japan lineage, China lineage and Laos/Thailand lineage, according to distribution and phylogenetic analysis of PDCoV. Among the 19 strains sequenced in this study, 12 strains had the most recent homology with Japanese strains, belonging to USA/Japan Lineage, and 7 strains had the most recent homology with Sichuan and Guangxi strains, belonging to China Lineage.

## Discussion

Under the pressure of African swine fever (ASF), the incidence of related diseases caused by porcine epidemic diarrhea virus (PEDV) and porcine delta-coronavirus (PDCoV) have also been increasing, causing substantial economic losses to the swine industry in China [13, 33]. PEDV and PDCoV can infect pigs of all ages [7, 34]. These two viruses not only cause diarrhea in pigs at different stages, but are also associated with porcine reproductive disorders and respiratory syndrome. Commercial vaccines for PEDV have been marketed and are now widely used in pig farms [35–39]. In the first few years of use, the vaccines offered good prevention and managed to control the viruses to a degree, but the immune effect of these vaccines has become poor in recent years [7, 8, 40, 41], which suggests that the epidemic strain of PEDV may have mutated. Therefore, there is an urgent need to develop and use a highly sensitive monitoring method for PEDV and PDCoV, to give early infection warning. In addition, it is equally important to clarify the molecular epidemiological background of viruses within positive samples and to provide that in a timely manner within the original testing method. To meet the above requirements, four pairs of primers were designed based on the nucleocapsid protein (N) gene fragments within PEDV and PDCoV, and the reaction conditions of the designed primers were optimized. The results showed that the dual-nested RT-PCR (dnRT-PCR) method established in this study exhibited good specificity for detecting the nucleocapsid protein (N) gene fragments of PEDV and PDCoV, and had no cross-reaction with PRRSV, TGEV, PoRV, PRV, E-coli or S. aureus. Repeatability and coincidence rate of this method were 100%. Electrophoresis results of the first PCR amplification showed that the detectable gene copy numbers of the outer primers were 1.2 × 10^2^ and 1.3 × 10^2^ respectively. The minimum detection limits of PEDV and PDCoV were 10 copies/ml respectively after two PCR amplifiers. This sensitivity was 10 times higher than that of the duplex RT-RPA method of PEDV and PDCoV established by Li G et al [42], The sensitivity of detection of both PEDV and PDCoV were also 10 times higher than observed using the multi-RT-PCR method against PEDV, TGEV, PoRV and PDCoV established by Jia, S et al., [43]. When compared with the LuminexxTAG multiple detection method of 11 diarrhea viruses established by Shi, Y et al [44], the sensitivity of the method established in this study was 100 times higher for both PEDV and PDCoV, indicating that among the methods currently reported, the sensitivity of the method established in this study was relatively high, it outperformed the other methods. The method also presented with good anti-interference, specificity and repeatability, therefore it is suitable for clinical or normal diagnostic detection and for early warning monitoring of both PEDV and PDCoV. Further, clinical follow-up was performed on pigs that tested negative using conventional RT-PCR but positive by dnRT-PCR, and the corresponding infected pigs showed clinical symptoms of diarrhea in varying degrees after 3–5 days. At this latter time point, both the dnRT-PCR and conventional RT-PCR methods indicated positive results, and sequencing further confirmed that the amplified specific fragments were positive. Therefore, this means a lot of time can be saved during the etiological investigation, costs can be reduced, and the current epidemic situation of PEDV and PDCoV in pig farms under pressure of ASF can be accurately evaluated.

A total of 251 samples were collected from 177 colostrum samples and 74 vulva samples. The positive rate of PDCoV in colostrum was 8.8% (22/251), which was higher than that of PEDV at 1.2% (3/251). This may suggest that PDCoV infects newborn piglets through sow milk. According to the scarce monitoring data available, the role of PDCoV in infecting newborn piglets through this pathway may be more important than that of PEDV. The detection rates of PEDV and PDCoV in vulva swabs were 2.4% (6/251) and 2.0% (5/251), respectively, indicating that a positive vulva swab means infection within the birth canal, which increases the risk of transvaginal infection, to the piglet, with both agents during pregnancy and birth. PEDV and PDCoV infections within the birth canal are also more likely to cause symptoms related to reproductive disorder in sows, this is consistent with the report published by Em-on Olanratmanee et al [45]. In addition, among the 251 samples submitted for examination, the positive rate of PEDV was 3.6% lower than that of PDCoV at 10.8%, which indicated that the diarrhea syndrome was more likely caused by PDCoV infection due to its higher infection rates. There were 8 dual infection samples within the positive samples, including 3 colostrum samples and 5 vulva samples, therefore, mixed infections of PEDV and PDCoV existed together in both colostrum and vulva samples. The probability of double infection within the vulva samples was higher than observed in colostrum, especially given that there were 177(1.7%) colostrum and 74(6.8%) vulva samples, but this further confirmed that the infection of these two viruses may be responsible for causing reproductive disorders in sows.

In this study, genetic evolution analysis of N gene of PEDV showed that the Chinese PEDV strains characterized in this study belong to G2, and they differed genetically from the vaccine strain (CV777) and the early Korean strains (SM98). In addition, according to the genetic evolution analysis of N gene of PDCoV, 12 sequencing strains belong to the USA/Japan lineage, and 7 sequencing strains belong to the China lineage, which indicates that the current American and Japanese strains of PDCoV have probably been widely circulated in China. The N gene was highly conserved but still had some unique point mutations as well as conserved regions. The establishment of dnRT-PCR method will contribute to the moecular epidemiology of PEDV and PDCoV in China and neighboring countries.

## Conclusion

This study established a highly sensitive method for early detection of PEDV and PDCoV, dnRT-PCR, which provided both convenience and an early warning of diarrheal diseases caused by these two viruses. This is of great significance for the sustainable development of the worldwide pig industry.

## Methods

### Viral and bacterial strains

Two viral strains were used as positive controls in this study: a cell cultured adapted “CV-777” strain of PEDV and a cell cultured adapted “CHN-HB” strain of PDCoV, which were identified and stored in our laboratory. To determine the specificity of our methods, the following viruses and bacteria were used: porcine reproductive and respiratory syndrome virus (PRRSV), swine transmissible gastroenteritis virus (TGEV), porcine rotavirus (PoRV), pseudorabies virus (PRV), Escherichia coli and Staphylococcus aureus, the above strains were also identified and stored in our laboratory.

### Sample collection and genome extraction

In November 2018, 251 samples of colostrum and vulva swabs of sows and newborn piglets were collected from large-scale pig farms in Hubei, Hunan, Sichuan and Guangdong province. Genomes from the control strains and clinical swabs were collected and centrifuged at 3000 r/min for 15 min [46]. Viral RNA was extracted from the samples to be tested using Trizol reagent (AXYGEN Bio., China) according to the manufacturer’s instructions, and the concentrations of the extracted RNA were measured for the evaluation of the duplex nested RT-PCR method developed in this study.

### Primer design

The nucleotide sequence for PEDV and the PDCoV were collected from GenBank database available in the National Center for Biotechnology Information server (http://www.ncbi.nlm.nih.gov/). According to the nucleotide sequence of the N gene, two pairs of specific primers for PEDV and two pairs of specific primers for PDCoV were designed using Primer 5(https://primer-premier-5.software.informer.com/). The conventional RT-PCR primers for PEDV and for PDCoV were designed according to the published reference [9] (Table 2).

**Table 2:** Primers designed for the duplex nested RT-PCR in this study

Virus	Target gene	Primer	Sequence	Position	Product size	Tm		Origin
PEDV	N	PEDV- O- F	5’-GCAAACGGGTGCCATTATCTC-3’	32–1027	995 bp		63.8	This paper
		PEDV- O- R	5’-GCTCACGAACAGCCACATTAC-3’				60.7	
		PEDV- I- F	5’-TTGGCATTTCTACTACCTCGGAACA-3’	216–956	740 bp		65.0	
		PEDV- I- R	5’-GCCTGACGCATCAACACCTTT-3’				64.7	
PDCoV	N	PDCoV- O- F	5’-CAGGTCCCAGAGGAAATCTTA-3’	218–883	665 bp		58.3	This paper
		PDCoV- O- R	5’-TTTGGTAGGTGGCTCATAGGT-3’				58.6	
		PDCoV- I- F	5’-TGCCAAACGCAACCC-3’	440–784	344 bp		58.5	
		PDCoV- I- R	5’-CAGCCATACCCGTCTTCT-3’				56.1	
PEDV	N	PEDV- F	5’-TAGGACTCGTACTGAGGGTGT-3’	26,642–26,662	600 bp		59.4	Ding et al.
		PEDV- R	5’-CTATTTTCGCCCTTGGGAATT-3’	27,222–27,242			56.6	
PDCoV	N	PDCoV- F	5’-GCTGACACTTCTATTAAAC-3’	24,301–24,319	497 bp		48.7	Ding et al.
		PDCoV- R	5’-TTGACTGTGATTGAGTAG-3’	24,804 − 24,787			48.4	

Note: PEDV-O-F, PEDV-O-R, PDCoV-O-F and PDCoV-O-R were the primers used in the first round of duplex nested RT-PCR; PEDV-I-F, PEDV-I-R, PDCoV-I-F and PDCoV-I-R were the primers used in the second round of duplex nested RT-PCR.PEDV- F, PEDV-R, PDCoV-F and PDCoV- R were the primers used for conventional RT-PCR.

### Construction of standard recombinant plasmids

The highly conserved N gene sequence of PEDV (CV-777 strain, Accession number: AF353511.1) was amplified using the PEDV-O-F and PEDV-O-R. The highly conserved N gene sequence of the PDCoV (CHN-HB strain, Accession number: KP757891.1) was amplified using the PDCoV-O-F, PDCoV-O-R. (The primers were highlighted in Table 2). The PCR product was cloned into the pMD-18 T vector (Takara, Dalian, China). The positive clone was selected and sequenced before further steps. Plasmid extraction was carried out with the MiniBEST Plasmid Purification Kit (Takara, Dalian, China) following the manufacturer’s instructions. DNA concentration was determined by spectro­photometry, and the copy number was calculated before use as a DNA standard for sensitivity analysis.

### Duplex nested RT-PCR

For the first reaction, the amplification was carried out in a total volume of 25 µl of reaction mixture containing 12.5 µl of 2 × 1 Step Buffer, 1 µl of PrimeScript 1 Step Enzyme Mix, 1 µl of PEDV-O-F, PEDV-O-R, PDCoV-O-F and PDCoV-O-R primers at the concentration of 20 µmol/L, respectively and 1 µl of RNA templet. RNase-free water was added bring the total volume to 25 µl. Amplification was performed with a thermocycler (Bioer Co., China) using the following procedure: 30 min at 50℃ for reverse transcription followed by predenaturation at 94 °C for 2 min, 32 cycles of denaturation at 94 °C for 30 s, annealing at 54 °C for 30 s, extension at 72 °C for 1 min, and a final extension at 72 °C for 8 min. The negative PCR product from the first reaction was diluted 50 times as the genetic template for the nested PCR.

For the nested PCR, the total volume of the reaction mixture was the same as the first reaction, but the reaction mixture contains 12.5 µl of PrimeScript 1 Step Enzyme Mix, 1 µl of PEDV-I-F, PEDV-I-R, PDCoV-I-F and PDCoV-I-R primers at the concentration of 20 µmol/L, respectively and 1 µl of templet. RNase-free water was added to bring the volume to 25 µl. The amplification procedure was as follows: predenaturation at 94 °C for 2 min, 32 cycles of denaturation at 98 °C for 30 s, annealing at 56 °C for 30 s, extension at 72 °C for 50 s, and a final extension at 72 °C for 8 min. The amplified products from two reactions were detected after electrophoresis on 1% agarose gels.

According to the results of the double nested RT-PCR amplification, the target band detected by electrophoresis after the first PCR amplification was reported to be positive (the 996 bp band detected was PEDV, and 665 bp was PDCoV); or double bands were detected, that is, both viruses were within the sample at the same time), and the sample was considered to have carried a high virulence load. Electrophoresis detected the target band after the second PCR amplification and was also judged to be positive (the 739 bp band was PEDV and the 344 bp band was PDCoV, or double bands were detected), but the virulence of the sample was relatively low. If no target bands were detected after two rounds of PCR, the samples were reported to be free of PEDV and PDCoV.

### Specificity and sensitivity of the dnRT-PCR primers

The pMD-18-T-PEDV and pMD-18-T-PDCoV positive recombinant plasmids were constructed, and their concentrations were 1.2 × 10^7^ copies/µL and 1.3 × 10^7^ copies/µL, respectively. The genomes of porcine reproductive and respiratory syndrome (PRRSV), porcine infectious gastroenteritis virus (TGEV), porcine rotavirus (PoRV), porcine pseudo-rabies virus (PRV), *Escherichia coli* and *Staphylococcus aureus* were extracted as templates. The four pairs of primers for PEDV-O and PEDV-I, PDCoV-O and PDCoV-I used in the double nested RT-PCR were tested for specificity to test the four pairs of primers. The positive plasmids obtained above were diluted to a single digit copy number by 10 times ratio, and plasmids with each dilution were selected as templates, and the PEDV-O and PDCoV-O primers were used for the first PCR amplification. The product of the first amplification served as templates for the corresponding gradient, and the PEDV-I and PDCoV-I primers were used for the second PCR amplification. The amplified products from the two reactions were detected following electrophoresis on 1% agarose gels. In addition, the sensitivity of the dnRT-PCR was compared with that of duplex RT-RPA [42].

### Interference test

The genome used in the specificity test was mixed with the same amount of the genome, and PEDV-O and PEDV-I, PDCoV-O and PDCoV-I primers were used for PCR amplification respectively to test the anti-interference of the primers and to confirm whether the target band could be amplified from the mixed genome template.

### Evaluation of the dnRT-PCR on clinical samples

To evaluate the reliability of the duplex nested RT-PCR on clinical specimens, a total of 251 samples of colostrum and vulva swabs from sows and newborn piglets were collected from Sichuan, Hunan, Hubei, Guangdong provinces and tested with this newly developed assay. The reliability of the detection results was evaluated via sequencing. In addition, the coincidence rate of the dnRT-PCR was compared with results from conventional RT-PCR.

### Homology analysis and genetic evolution tree analysis

The sequencing results were analyzed using DNAstar Megalign Software (MegAlign Pro | Sequence Alignment Software | DNASTAR) and MEGA 7.0 Software (MEGA 7.O). The evolutionary tree was plotted by neighbor-joining method 500 times. The PEDV and PDCoV reference strains are shown in Tables 3 and 4, respectively. The sequences of PEDV and PDCoV generated from this study are shown in Supplementary 1 and Supplementary file 2, respectively.

**Table 3:** Reference strains for the homology analysis based on the N gene sequences of PEDV

Strain	Source	GenBank
CV777	Switzerland	AF353511.1
FR/001/2014	France	KR011756
USA/Indiana/17,846/2013	USA	KF452323.1
GXGG07	China	MK731895.1
JS-HZ2012	China	KC210147.1
SM98	Korean	GU93779
CH/YNKM-8/2013	China	KF761675.1
LZC	China	EF185992

**Table 4:** Reference strains for the homology analysis based on the N gene sequences of PDCoV

Strain	Source	GenBank
GX03-2019	China	MT648385.1
CHN/Sichuan/2019	China	MK993519.1
USA/Ohio444/2014	USA	KR265862
USA/Iowa459/2014	USA	KR265865
OH1987	USA	KJ46246
YMG/JPN/2014	Japan	KU051641.1
PDCoV/Swine/Thailand/S5015L/2015	Thailand	KU051649
P1_16_BTL_0115/PDCoV/2016/Lao	Lao	KX118627

### Electronic supplementary material

Below is the link to the electronic supplementary material.

**Additional file 2**: The sequences of PDCoV generated from this study.

**Additional file 1**: The sequences of PEDV generated from this study

## Data Availability

All data generated or analysed during this study are included in this published article. All the nucleotide sequences generated from this study have been deposited and are available in the GenBank database.

## List of abbreviations

PEDV: Porcine epidemic diarrhea virus
PDCoV: Porcine deltacoronavirus
dnRT-PCR: Duplex nested RT-PCR
